# Atypical secondary syphilis presentation in a patient with human immunodeficiency virus infection: a case report

**DOI:** 10.1186/s13256-019-2291-5

**Published:** 2019-12-09

**Authors:** Nina Yancheva, Elena Petrova, Tatyana Tchervenyakova

**Affiliations:** 1 Department of Infectious Diseases, Parasitology, and Tropical Medicine, Medical University – Sofia, Sofia, Bulgaria; 2 Department for AIDS, Specialised Hospital for Active Treatment of Infectious and Parasitic Diseases, 17 Akademik Ivan Geshov Blvd., 1303 Sofia, Bulgaria; 3 Department of Dermatology, Medical University – Sofia, Sofia, Bulgaria

**Keywords:** Syphilis, HIV, Atypical presentation, Misdiagnosis

## Abstract

**Introduction:**

Untreated syphilis may lead to severe complications. This infection has recently re-emerged in developed countries with a high number of cases coinfected with human immunodeficiency virus. In these patients, the skin lesions of secondary syphilis can be very atypical.

**Case presentation:**

We report the case of a 38-year-old Bulgarian homosexual man who was coinfected with human immunodeficiency virus and syphilis. His skin contained multiple extensive necrotic lesions with abundant purulent secretion that covered his face, lips, scalp, and torso. Initial clinical diagnoses included varicella pustulosa and staphylococcal dermatitis. Human immunodeficiency virus infection in our patient had been established 2 years earlier in prophylactic studies, but had not been treated. Due to lack of penicillin, he was successfully treated with ceftriaxone, and the skin lesions underwent complete reversal. He also began antiretroviral therapy, which resulted in a significant effect on his immune status. Three months after the onset of antiretroviral therapy, he also achieved optimal viral suppression.

**Conclusion:**

This case emphasizes the importance of considering cutaneous secondary syphilis in the differential diagnosis of any inflammatory cutaneous disorder in individuals infected with human immunodeficiency virus.

## Introduction

Syphilis has been known as “the great imitator” due to its wide variability in clinical presentation [1, 2]. The primary stage of the infection is classically defined by an asymptomatic chancre at the inoculation site [1]. The secondary stage results from the systemic dissemination of the infection, and is typically characterized by cutaneous eruptions, regional lymphadenopathy, and flu-like symptoms. Secondary syphilis classically features a copper-colored maculopapular rash with sharply delineated margins presenting typically on the palmar and plantar surfaces [1]. Verrucous lesions appearing as moist exophytic plaques on the genitals, intertriginous areas, and/or perineum have also been described, and are referred to as condyloma lata in the setting of secondary syphilis [1]. The secondary stage is followed by an asymptomatic latent period that may last months to years, followed by the tertiary stage, which is characterized by the neurologic, cardiovascular, and/or gummatous manifestations that lead to the high percentage morbidity and mortality associated with syphilis. Untreated syphilis may lead to severe complications [1]. The infection has recently re-emerged amid the homosexual population in developed countries with a high number of cases coinfected with human immunodeficiency virus (HIV) [3, 4]. Syphilis has been reported to assume uncommon clinical appearances, especially in patients infected with HIV type 1 [5]. A rare form of the disease is malignant syphilis, which is a form of destructive syphilid, with ulcerative lesions and severe toxemia that may have a lethal outcome [6, 7]. The ulcers, commonly seen over the face and the extremities, are covered with thick crusts, which heal slowly [6, 7]. The mucous membranes of the mouth and the nose may be involved, and prodroma such as fever, myalgia, and headache are common [1, 6, 7]. These lesions were frequently reported in the pre-antibiotic era, and have now re-emerged with the advent of HIV.

The case we present is unusual in the severity and the characteristics of the skin lesions that were not typical of secondary syphilis. Although the patient had consulted an experienced dermatologist in out-patient settings, initially, a syphilis diagnosis was not established and the patient was treated for staphylococcal dermatitis and pustular varicella. We would like to emphasize that syphilis can present extremely atypically in patients coinfected with HIV and syphilis, even in cases in which the patient is not yet in the stage of acquired immunodeficiency syndrome (AIDS).

## Case presentation

**Fig. 1:**
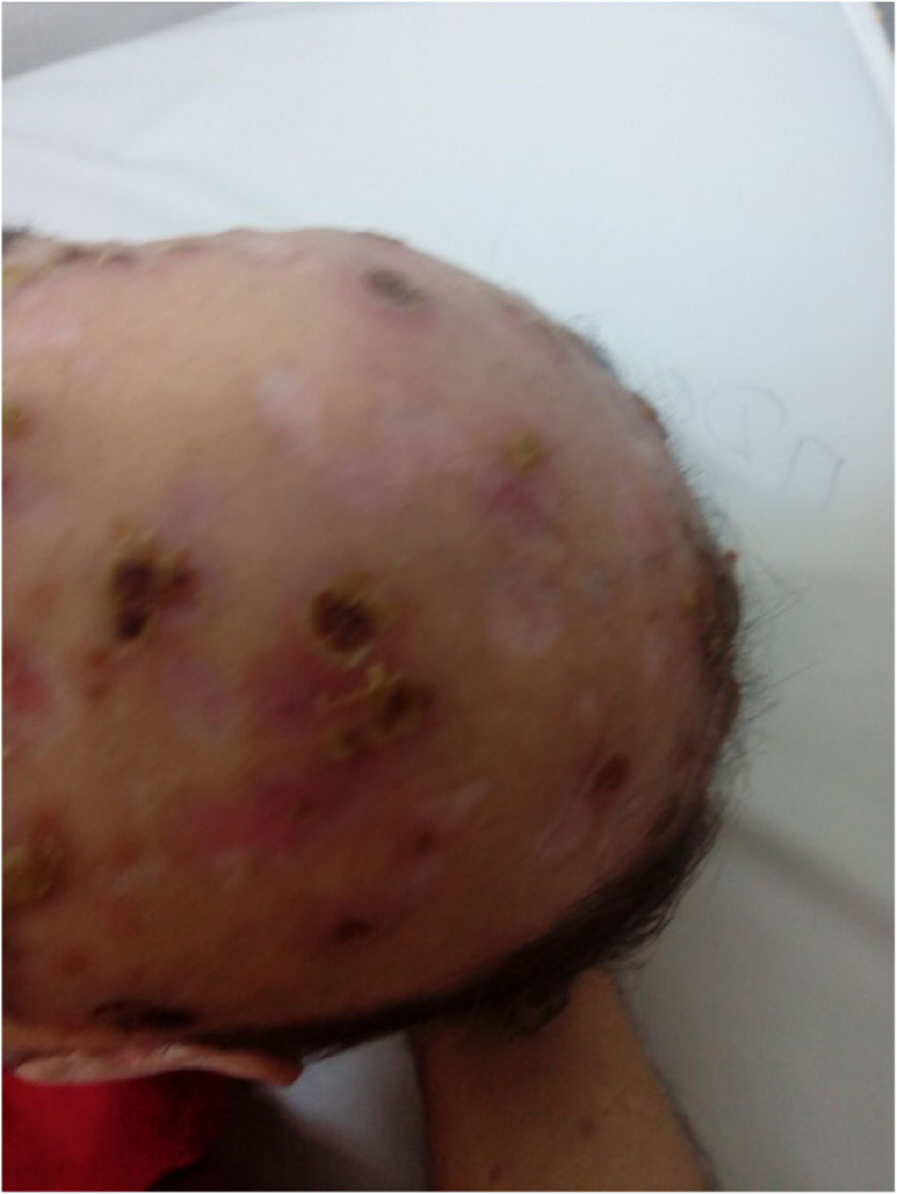
Skin lesions on the patient’s head

**Fig. 2:**
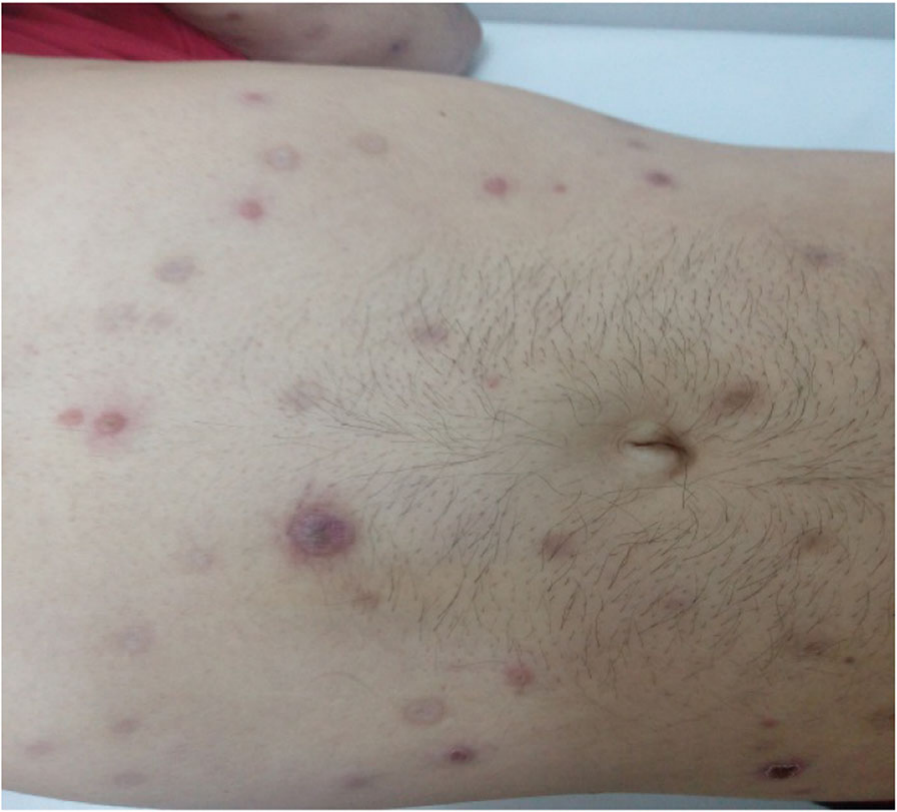
Skin lesions on the patient’s torso

**Fig. 3:**
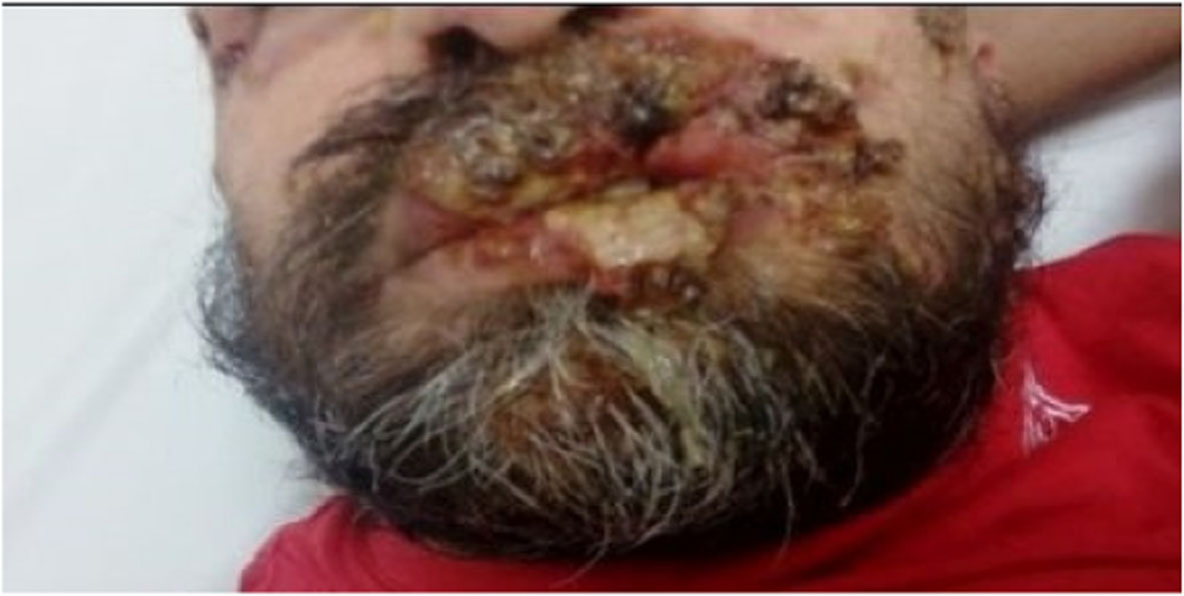
Skin lesions on the patient’s lips

We present the case of a 38-year-old Bulgarian homosexual man who was HIV-seropositive. According to his own account, he had several sexual partners and worked as a physical therapist. He neither smoked tobacco nor drunk alcohol; he actively practiced Taekwondo. He came to our Department with fever, chills, malaise, and multiple cutaneous lesions with purulent secretion that covered his face, scalp, and body. The skin lesions had appeared several months prior to a diagnosis. He explained that the initial lesions had looked like “pimples,” but subsequently had become “bubbles” filled with pus. Initial clinical diagnoses had included varicella pustulosa and staphylococcal dermatitis. He had been treated in out-patient settings with orally administered acyclovir 5 × 800 mg for 7 days and amoxicillin-clavulanic acid 3 × 1.0 g for 10 days. After an outbreak of fever and rash for approximately a week, his complaints had reappeared with more pronounced intensity and had continued for 2 months until a diagnosis was established. Following an out-patient visit to another dermatologist, he had been referred for syphilis and HIV testing. Specifically, the rapid plasma reagin (RPR) test and the *Treponema pallidum* particle agglutination assay (TPPA) had turned positive. It was established that 2 years earlier in prophylactic studies, he had been diagnosed as having an HIV infection and his result had been confirmed by the National Reference Laboratory for HIV in Sofia, Bulgaria. He claimed that he had not been aware of the diagnosis and had not been treated for it. He was therefore hospitalized at the Department for AIDS. A physical examination revealed extensive necrotic lesions on his face and head (Fig. 1) with abundant purulent secretion in the absence of lesions on his palmar and plantar surfaces, as well as the described papular lesions on his torso (Fig. 2) and, more scarcely, on his extremities. The most significant necrotic lesions occurred on his lips, and those produced the most purulent secretion (Fig. 3). He was febrile, and had lymphadenopathy and oropharyngeal candidiasis.

The physical examination on admission found fever of up to 39 ºC and generalized lymphadenomegaly. His breathing was clear, without any wheezing. His heart rate was 123 beats per minute and his blood pressure was 130/100 mmHg. No hepatosplenomegaly was detected. A neurological examination established no pathological findings.

The laboratory work showed evidence of anemia with hemoglobin (Hb) of 117 g/l and high leukocytosis with white blood cells (WBC) of 18.8 G/l and granulocytosis with granulocytes (Gran) of 91%. The biochemical tests were also normal: alanine aminotransferase (ALAT), 39 U/l; aspartate aminotransferase (ASAT), 32 U/l; glucose, 4.05 mmol/l; creatinine, 74.73 μmol/l; and total cholesterol, 2.8 mmol/l. Urine analysis revealed the following: pH, 6.0; protein, negative; glucose, negative; and bilirubin, negative.

Immune status tests showed a CD4 cell count of 208 cells/μl, a CD8 count of 845 cells/μl, and a CD4/CD8 ratio of 0.20. The viral load for HIV was 745,000 copies/μl.

Microbiological examinations were performed on throat secretions, nasal secretions, sputum, uroculture, and hemoculture. The samples were collected and transported in sterile conditions. Seeding of media for aerobic and anaerobic microorganisms was done. The only isolated microorganism was *Candida albicans* in non-significant amounts in sputum.

An X-ray of his lungs and heart revealed no pathological findings.

A skin biopsy was performed, and a histological examination showed non-specific inflammatory changes: a moderate mixed inflammatory infiltrate located perivascularly throughout the dermis, which was composed of lymphocytes, plasmacytes, and polymorphonuclear cells.

**Fig. 4:**
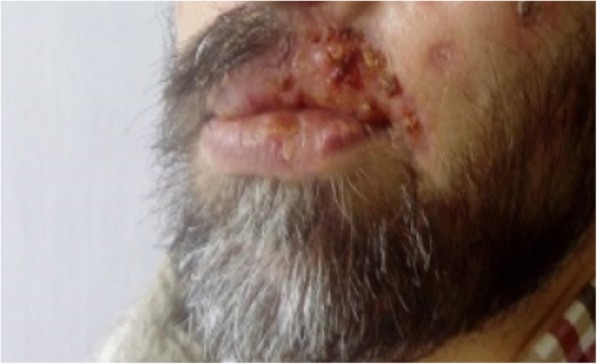
The patient in the first month after treatment

Due to a lack of penicillin, our patient was given a 21-day course of ceftriaxone 2 × 2.0 g administered intravenously in combination with amikacin 2 × 500 mg administered intravenously and metronidazole 3 × 500 mg administered intravenously, parallel to the administration of an antimycotic agent, fluconazole 200 mg administered intravenously, for the oropharyngeal candidiasis. The idea was to cover all possible superposed infections with Gram-negative and anaerobic bacteria, too. Our patient also began antiretroviral therapy with the following combination: emtricitabine/tenofovir disoproxil fumarate 0.445 + dolutegravir 0.050. A month after the onset of treatment, he was discharged with an almost complete reversal of the skin lesions (Fig. 4). His upper lip was seen to be retracted after the reversal of the necrotic lesions (Fig. 4). Three months later, an RPR test was negative, the CD4+ T count was elevated (to 522 cells/μl), and so was the CD4/CD8 ratio (0.58), while the viral load became undetectable (< 40 copies/μl).

Unfortunately, as of May 2019, our patient has not visited our Department, and has not been followed up since then.

## Discussion

We present a case of a patient with secondary syphilis and coinfection with HIV. The diagnosis was delayed by several months, leading to severe skin lesions, which subsequently led to facial deformity.

Secondary syphilis refers to the stage of *Treponema pallidum* infection at which the greatest load of spirochetes is present in the bloodstream and many other tissues [1]. Skin lesions are the most common manifestation and can include macular, maculopapular, nodular, pustular, and papulosquamous rash, and a mix of all types [1]. According to many authors, HIV- syphilis coinfection can be associated with the following: multiple or deeper chancres; overlapping of features of primary and secondary syphilis; more rapid progression to tertiary syphilis; ocular syphilis; false negative serology; clinically important neurological disease; and lesser efficacy of the standard therapy for early syphilis. The most frequent clinical manifestation of secondary syphilis in this group is the maculopapular rash [8, 9]. In our case, most of the lesions were pustules and ulcerations with abundant purulent secretion in the absence of lesions on the palmar and plantar surfaces. Therefore, our patient may also be considered to have an atypical form of syphilis. The abundant purulent secretion from some of the lesions, especially on the lips, is uncommon. We have not observed such skin lesions in a patient coinfected with HIV and syphilis over the past 10 years. The interaction between syphilis and HIV infection is complex, and has not been understood completely. Through its modulation of the immune response, HIV can affect the course, evolution, diagnosis, and response to the treatment of syphilis [10]. The course of secondary syphilis as “malignant,” that is, with necrotic lesions with purulent secretion, is probably due to the underlying untreated HIV infection with a high viral load and advanced immune deficiency.

Treatment for secondary syphilis includes 2.4 million units of benzathine penicillin G administered intramuscularly in a single shot, while latent syphilis requires intramuscular administration of 2.4 million units of benzathine penicillin G once a week for 3 weeks [10, 11]. According to a meta-analysis comparing the efficacy of ceftriaxone to penicillin in the treatment of syphilis, there is no evidence in the literature that ceftriaxone is less efficient than penicillin [10, 11]. In our case, as benzathine penicillin G is not available in Bulgaria for the time being, our patient was successfully treated with a prolonged course with ceftriaxone.

## Conclusions

Secondary syphilis in patients with HIV infection may resemble varicella pustulosa, staphylococcal dermatitis, or cutaneous lymphoma. This can lead to delayed diagnosis and severer skin lesions, as demonstrated by our case, which emphasizes the importance of considering cutaneous secondary syphilis in the differential diagnosis of any inflammatory cutaneous disorder in individuals with HIV infection. Both diseases are common sexually transmitted infections among men who have sex with men. This indicates that that particular risk group must be actively monitored for these infections.
